# Beyond the Apex: A Case Series of Mid-ventricular Takotsubo Cardiomyopathy

**DOI:** 10.7759/cureus.102063

**Published:** 2026-01-22

**Authors:** Fnu Parul, Karuna Rayamajhi, Rohan Kumar, Akhil Gaderaju, Mahmoud Khairy, Appa Bandi

**Affiliations:** 1 Internal Medicine, University of Michigan Health-Sparrow Hospital, Michigan State University, East Lansing, USA; 2 Medicine, Pandit Bhagwat Dayal Sharma Post Graduate Institute of Medical Sciences, Rohtak, IND; 3 Cardiology, University of Michigan Health-Sparrow Hospital, Michigan State University, East Lansing, USA

**Keywords:** atypical takotsubo cardiomyopathy, broken-heart syndrome, mid-ventricular ballooning syndrome, stress-related cardiomyopathy, takotsubo cardiomyopathy (ttc)

## Abstract

Takotsubo cardiomyopathy (TCM), also known as stress-induced cardiomyopathy, is characterized by transient, reversible left ventricular systolic dysfunction in the absence of obstructive coronary artery disease (CAD). It can mimic acute coronary syndromes, posing diagnostic challenges that require a high index of suspicion. We present two cases of mid-ventricular TCM, each triggered by distinct acute physical stressors. The first case involves a 63-year-old male with a history of cerebrovascular accident and nicotine dependence who presented following a generalized tonic-clonic seizure. His electrocardiogram demonstrated ST-segment elevations in the inferolateral leads, and laboratory workup revealed elevated cardiac biomarkers. Coronary angiography showed mild non-obstructive CAD, while echocardiography revealed mid-ventricular regional wall motion abnormalities with an ejection fraction (EF) of 35-40%. He was treated with beta-blockers, angiotensin receptor blockers, aspirin, and statin therapy, with complete recovery of cardiac function on follow-up. The second case involves a 66-year-old female with a history of asthma, obstructive sleep apnea, and prior breast cancer who developed acute hypoxic respiratory failure following hashish inhalation. She presented with chest pain and elevated troponin levels, but without ST-segment elevation. Echocardiography showed mid-ventricular wall motion abnormalities and a reduced EF of 30-35%, while coronary angiography demonstrated no significant obstructive disease. Her presentation was consistent with mid-ventricular TCM secondary to acute hypoxemia and physical stress. These cases highlight the diagnostic variability and clinical spectrum of TCM, emphasizing the need for early recognition of atypical variants. Awareness of mid-ventricular presentations is essential for prompt diagnosis, appropriate management, and prevention of recurrence in patients presenting with acute cardiac dysfunction without significant coronary obstruction.

## Introduction

Takotsubo cardiomyopathy (TCM), also known as stress-induced cardiomyopathy, is characterized by a temporary and reversible regional left ventricular systolic dysfunction that mimics an acute coronary event, despite the absence of obstructive coronary artery disease (CAD) [1]. It represents approximately 2% of cases within the ST-elevation myocardial infarction (STEMI) population and predominantly affects postmenopausal women, who account for about 90% of all cases [2]. This condition is typically triggered by sudden, severe emotional or physical stress, which leads to excessive catecholamine release, resulting in microvascular dysfunction and intracellular calcium overload [3].

Clinically, TCM often presents with akinesis or hypokinesis of the apical segment of the left ventricle, commonly with hyperkinetic basal segments. However, atypical variants involving regional wall motion abnormalities have been reported, including basal, focal, mid-ventricular, biventricular (involving both the apical and right ventricle), isolated right ventricular, and even global variants [4]. Although apical form remains the most common, the diagnosis of midventricular variant has increased from 18% to 28% from 2004 to 2021 [5]. Recognition of the mid-ventricular subtype of Takotsubo syndrome is clinically important, as this form may be associated with additional complications, including dynamic left ventricular outflow tract (LVOT) obstruction, alongside an increased risk of hemodynamic compromise and cardiogenic shock. Furthermore, compared with the classic apical variant, the mid-ventricular subtype may exhibit more subtle and evolving electrocardiographic changes, which can delay or complicate diagnosis, as previously reported in the literature [6].

## Case presentation

Case 1

**Figure 1:**
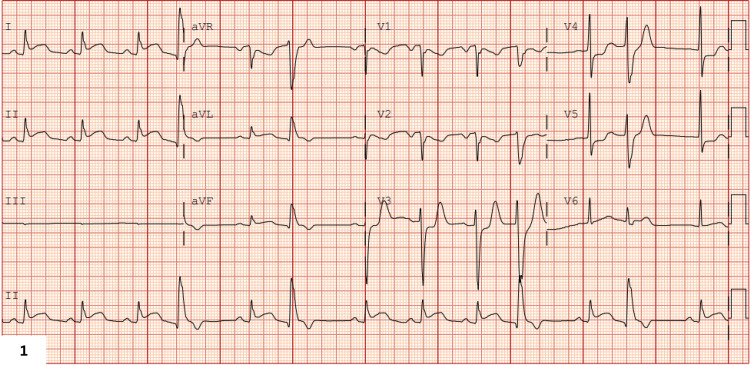
On-admission electrocardiogram showing ST-segment elevation in inferior and lateral leads consistent with acute myocardial infarction

A 63-year-old male with a medical history of cerebrovascular accident and nicotine dependence presented to the emergency department in a post-ictal, confused state following a generalized tonic-clonic seizure. On admission, the cardiopulmonary examination revealed normal heart sounds and mild bibasilar crackles. His vital signs were within normal limits (heart rate (HR) of 80-90 bpm, blood pressure (BP) of 128/64 mmHg, and SpO_2_ of 94% on room air), except for mild tachypnea with a respiratory rate of 22 bpm. ECG demonstrated ST elevations in the inferior and lateral leads (Figure 1). Initial laboratory evaluation revealed elevated lactate, ammonia, high-sensitivity troponin I, and creatine phosphokinase (CPK) levels (Table 1).

**Table 1:** Significant laboratory findings on admission (Case 1)

Variables	Value	Reference Range
Lactate	13.3 mmol/L	0.2-1.8 mmol/L
Ammonia	124 µmol/L	13-37 umol/L
High-sensitivity troponin I	34->7334->8522 ->5334 ->3005 ng/L	0-18 ng/L
CPK (creatine phosphokinase)	726U/L	0-200 U/L

A bedside ultrasound showed a reduced ejection fraction (EF) of 30%. The patient underwent emergent cardiac catheterization, which demonstrated non-obstructive CAD, with patent left main and left anterior descending arteries, mild luminal irregularities in the left circumflex artery with approximately 30% proximal stenosis, and a dominant right coronary artery with moderate (40-50%) proximal stenosis. Left ventriculography revealed reduced systolic function with an estimated EF of 25-30% and mid-ventricular wall motion abnormalities. However, he developed cardiogenic shock, requiring vasopressor support and a brief admission to the intensive care unit. Notably, the patient did not report any subsequent chest pain, and his troponin levels gradually decreased. Echocardiography done during hospitalization revealed regional wall motion abnormalities (RWMAs) of the mid-ventricular segments with an EF of 35-40% (Videos 1-2). 

**Video 1:** Trans-thoracic echocardiography apical four-chamber view Trans-thoracic Echocardiography apical four-chamber demonstrating a mid-ventricular variant of takotsubo cardiomyopathy, with akinesis involving the mid-lateral and mid-septal segments, with normal apical motion and hyperkinetic basal segment.

**Video 2:** Trans-thoracic echocardiography contrast-enhanced view Trans-thoracic echocardiography contrast-enhanced view re-demonstrating a mid-ventricular variant of takotsubo cardiomyopathy, with akinesis involving the mid-lateral and mid-septal segments, with normal apical motion and hyperkinetic base.

In the absence of obstructive disease, a setting of acute physical stress (seizure), and RWMAs, the patient was diagnosed with a mid-ventricular variant of takotsubo cardiomyopathy. He was initiated on aspirin, statin, beta-blocker, and angiotensin receptor blocker and discharged in a hemodynamically stable position. On follow-up three months after discharge, the patient was found to be doing well, with the repeat echocardiogram showing improvement in ejection fraction to 60% and resolution of RWMAs.

Case 2

A 66-year-old female with a medical history of moderate persistent asthma, obstructive sleep apnea, alpha-1-antitrypsin deficiency, and prior right breast cancer treated with lumpectomy, chemotherapy, and radiation presented to the emergency department with acute shortness of breath following hashish inhalation. Her symptoms were accompanied by central chest pain radiating to the left arm, jaw, and neck.

**Figure 2:**
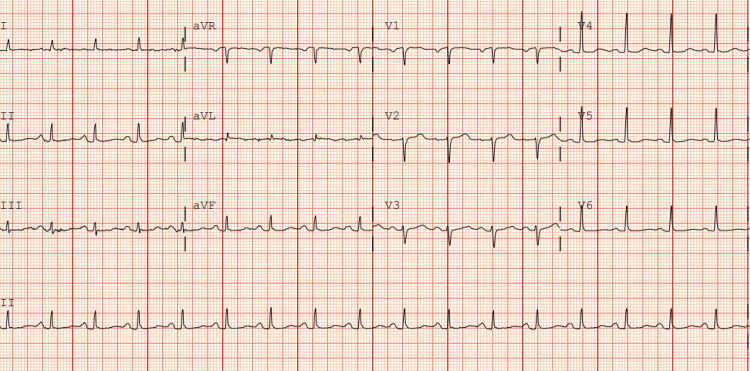
On-admission electrocardiogram showing sinus tachycardia with no significant changes consistent with acute ischemia

On admission, she was tachycardic (HR of 110-115), tachypneic (RR of 24-28), hypertensive (BP of 150/90-160/100), and hypoxemic, with oxygen saturation falling to 80% on room air. Examination revealed bilateral expiratory wheezing in the lungs with otherwise normal heart sounds. Chest radiograph showed no acute cardiopulmonary findings, and the initial ECG did not demonstrate ST-segment elevations (Figure 2). Arterial blood gas analysis demonstrated acidemia. Laboratory studies revealed leukocytosis, mildly elevated CRP, and elevated high-sensitivity troponin I levels, peaking at 717 ng/L (Table 2). Further, chest computed tomography with contrast showed no evidence of pulmonary embolism or other significant abnormalities, except for mild emphysematous changes.

**Table 2:** Significant laboratory findings on admission (Case 2)

Variables	Value	Reference Range
Arterial blood pH	7.29	7.35-7.45
White blood cells	23,900 /µL	4.0-12.0 10*3/uL
High-sensitivity troponin I	74 -> 382 ->610 ->717 ->457 ng/L	0-18 ng/L

She was managed for acute hypoxic respiratory failure with noninvasive positive-pressure ventilation, intravenous corticosteroids, antibiotics, magnesium sulfate, and inhaled long-acting muscarinic (LAMA) and β-adrenergic (LABA) agents. Given concern for a non-ST elevation myocardial infarction, she received aspirin and intravenous heparin. A transthoracic echocardiogram revealed RWMAs involving the mid-ventricular segments of the left ventricle, with a reduced ejection fraction of 30-35% (Videos 3-4). In view of rising troponins and new systolic dysfunction, she underwent left heart catheterization, which demonstrated no significant obstructive coronary artery disease. Left ventriculography revealed reduced systolic function with an estimated ejection fraction of 30-35% and mid-ventricular wall motion abnormalities.

**Video 3:** Trans-thoracic echocardiography apical two-chamber view Trans-thoracic echocardiography apical two-chamber imaging demonstrates a mid-ventricular variant of stress cardiomyopathy (takotsubo), characterized by akinesis of the mid-anterior and mid-inferior walls, with sparing of the basal and apical segments.

**Video 4:** Trans-thoracic echocardiography contrast-enhanced view Trans-thoracic echocardiography contrast-enhanced apical four-chamber view demonstrating a mid-ventricular variant of takotsubo cardiomyopathy, with akinesis involving the mid-lateral and mid-septal segments, while apical and basal segments exhibit normal systolic function.

In the absence of ischemic coronary pathology and in the setting of acute physical stress due to hypoxemia, the presentation was consistent with a mid-ventricular variant of stress (takotsubo) cardiomyopathy. She was initiated on aspirin, statin, beta-blocker, and angiotensin receptor blocker and discharged in a hemodynamically stable position. On one-month follow-up, she continued to recover well with no residual symptoms. Her repeat echocardiography done on follow-up demonstrated resolution of RWMAs with normalisation of ejection fraction to 60-65%.

## Discussion

TCM, also known as “broken heart syndrome,” is estimated to affect approximately 1-3% of patients presenting with symptoms of acute coronary syndrome (ACS), with the majority of cases occurring in women, particularly postmenopausal women [2]. Although the prevalence is lower in younger age groups, it has been observed to affect more males in this population, especially those with fewer comorbidities or with acute neurological or psychiatric disorders. In such cases, it often presents as atypical variants or as an in-hospital complication [7]. Although apical form remains the most common, the diagnosis of midventricular variant has increased from 18% to 28% from 2004 to 2021 [5]. Patients with this variant typically exhibit mid-ventricular wall motion abnormalities with hyperdynamic apical and basal segments.

Several theories have been proposed regarding the pathogenesis of TCM, with the adrenergic hypothesis being the most widely accepted. In response to severe acute emotional or physical stress, excessive catecholamines are released, resulting in microvascular dysfunction and intracellular calcium overload, further causing catecholamine-induced myocardial damage [3].

The diagnosis of TCM is based on clinical presentation, laboratory findings, and diagnostic imaging. The revised Mayo Clinic diagnostic criteria, which include (a) transient dyskinesis of the left ventricular (LV) mid-segments, (b) regional wall motion abnormalities extending beyond a single epicardial vascular distribution, (c) absence of obstructive CAD or acute plaque rupture, and (d) new electrocardiographic changes or modest troponin elevation, along with the exclusion of pheochromocytoma and myocarditis, is used for establishing the diagnosis [8]. Although it typically mimics ACS, with ischemic changes on the electrocardiogram and elevated troponin levels (as seen in the case presented here), coronary angiography typically reveals no significant obstructive coronary disease [1]. However, co-existing CAD may be present in approximately 15% of patients [9,10]. In such cases, careful correlation between angiographic findings and wall motion abnormalities is necessary. Additional assessments, such as optical coherence tomography and intravascular ultrasound, can help evaluate plaque rupture, which is not characteristic of TCM [11]. Echocardiography is a valuable tool for assessing the location, extent, and severity of RWMAs and detecting complications. Cardiac magnetic resonance imaging (MRI) with gadolinium contrast can further differentiate takotsubo syndrome from acute myocardial infarction and myocarditis by demonstrating an absence of fibrosis, which is consistent with TCM [12].

Follow-up management of TCM focuses on potential relapse due to the recurrence of stressors and resolution of RWMAs, in addition to monitoring for the development of comorbidities and CAD risk factors. Repeat echocardiograms within a few weeks or cardiac MRI when necessary are often used for this purpose. Treatment aims to prevent recurrence, alleviate symptoms, and manage complications. Current protocols recommend using beta-blockers, renin-angiotensin-aldosterone system inhibitor (RAASi), and statins to reduce myocardial stress induced by excessive catecholamine release [13].

Given the risk for the development of complications, the most common of which include LVOT obstruction secondary to hyperdynamic basal segments and cardiogenic shock, the potential for reversibility, and atypical presentations, early diagnosis is crucial for TCM. Prompt and accurate identification allows for effective management of complications and prevention of syndrome recurrence.

## Conclusions

We present a case series of mid-ventricular TCM, an uncommon variant of stress-induced cardiomyopathy, triggered by distinct forms of acute physical stress-seizure and hypoxemia. These cases underscore the heterogeneous presentations and multifactorial triggers associated with this atypical subtype. Early recognition, guided by clinical suspicion and confirmed through echocardiography and coronary angiography, is critical for accurate diagnosis and management. Awareness of mid-ventricular variants can prevent misdiagnosis, unnecessary invasive procedures, and ensure timely initiation of supportive therapy, leading to favorable outcomes.
